# Circulating Sestrin Levels Are Increased in Hypertension Patients

**DOI:** 10.1155/2020/3787295

**Published:** 2020-06-12

**Authors:** Cao Fang, Zicong Yang, Lei Shi, Tao Zeng, Ying Shi, Ling Liu, Hongtao Liu, Yingzhong Lin

**Affiliations:** ^1^Xishui County People's Hospital Affiliated to Hubei University of Science and Technology, Hubei 530021, China; ^2^Department of Cardiology, The People's Hospital of Guangxi Zhuang Autonomous Region, Nanning 530021, China; ^3^Department of Cardiovascular Medicine, Shenzhen Longhua District Central Hospital, Longhua Central Hospital Affiliated to Guangdong Medical University, Shenzhen, Guangdong Province, 518110, China

## Abstract

**Background:**

Sestrins (Sesns), a group of oxidative stress-related proteins, have been reported to be involved in various cardiovascular diseases, including aortic dissection and chronic heart failure. This study is aimed at investigating the level of circulating Sesn1, Sesn2, and Sesn3 in hypertension patients.

**Methods:**

Plasma levels of Sesn1, Sesn2, and Sesn3 in 400 hypertensive patients and 100 normotensive subjects were detected using enzyme-linked immunosorbent assay (ELISA) kits. The hypertension patients were divided into groups with grade I (*n* = 140), grade II (*n* = 180), and grade III (*n* = 80) hypertension.

**Results:**

Compared with the normotensive subjects, Sesn1, Sesn2, and Sesn3 levels were increased in patients with hypertension, with a gradual increase between the groups with grade I, grade II, and grade III hypertension. Elevated Sesn1, Sesn2, and Sesn3 levels were positively correlated with both the systolic blood pressure (SBP) and diastolic blood pressure (DBP). Moreover, Sesn1, Sesn2, and Sesn3 levels were elevated in patients with dipper hypertension and further increased in patients with nondipper hypertension. In addition, smokers, as well as patients with higher levels of angiotensin II (Ang II) and carotid atherosclerotic plaque (CAP), exhibited increased Sesn1, Sesn2, and Sesn3 levels when compared with patients without these clinical characteristics. Furthermore, plasma levels of Sesn1, Sesn2, and Sesn3 were negatively correlated with the presence of CAP.

**Conclusions:**

Circulating Sesn levels are increased in patients with hypertension and may be a target for the prevention and treatment of clinical hypertension.

## 1. Introduction

Hypertension is the most common risk factor of cardiovascular disease, leading to significantly higher morbidity and mortality than other risk factors. Numerous previous studies have used the immune effects and inflammatory response of blood vessels to strengthen the pathogenesis of hypertension [1, 2]. In recent years, more and more studies have proved that oxidative stress was closely related to the progression of hypertension [3–5].

Sestrin (Sesn) family members, including Sesn1, Sesn2, and Sesn3 in mammals, are proteins that regulate oxidative stress both *in vivo* and *in vitro* [6, 7]. Sesn is widely present in a variety of tissues and organs, and its expression is majorly regulated by oxidative stress levels and, to a lesser extent, by inflammatory responses, aging, and other pathophysiological effects [8–10]. A number of studies have confirmed that Sesns are involved in the development and progression of various diseases, by regulating oxidative stress. Moreover, some studies have reported that Sesns can also regulate inflammation, apoptosis, aging, and autophagy [9–13].

Sesn1, Sesn2, and Sesn3 were also shown to play a critical role in cardiovascular diseases [14]. Elevated plasma Sesn1, Sesn2, and Sesn3 levels were observed in patients with coronary artery disease (CAD). Furthermore, they were positively correlated with malondialdehyde (MDA) levels and negatively correlated with superoxide dismutase (SOD) levels [15]. Knockdown of Sesn1 significantly alleviated phenylephrine- (PE-) induced cardiac hypertrophy by regulating the AMPK/mTORC1/autophagy axis, while Sesn1 overexpression led to the opposite effect [16]. Expression of circulating Sesn2 was shown to be increased in aortic dissection and chronic heart failure (CHF) patients. Moreover, CHF patients with elevated Sesn2 levels exhibited poor outcomes and more cardiac events [17, 18]. Knockout of Sesn2 enlarged the area of myocardial infarction after ischemia-reperfusion and aggravated myocardial injury after radiation [19, 20]. In addition, Sesn2 protected against age-related intolerance to postmyocardial infarction and phenylephrine- (PE-) induced cardiomyocyte hypertrophy [21, 22]. However, the current expression levels of circulating Sesns in patients with hypertension are still unknown. Therefore, this study is aimed at examining plasma Sesn1, Sesn2, and Sesn3 levels in hypertension patients.

## 2. Methods

### 2.1. Study Populations

In the present study, 100 normotensive and 400 primary hypertension patients were enrolled. The patients diagnosed with diseases, including secondary hypertension, chronic heart failure, chronic renal failure, stroke, valvular heart disease, collagen disease, advanced liver disease, malignant disease, septicemia, or other diseases that may affect Sesn expression, were excluded from this study. According to the value of systolic blood pressure (SBP) and/or diastolic blood pressure (DBP), the hypertension patients were divided into three groups, namely, grade I hypertension (*n* = 140), grade II hypertension (*n* = 180), and grade III hypertension (*n* = 80). All subjects underwent ambulatory blood pressure monitoring to determine the type of hypertension and were further divided into two groups, namely, the scoop-shaped hypertension group (*n* = 240) and nonscoop-shaped hypertension group (*n* = 160). In addition, all subjects received a carotid ultrasound to determine the presence of carotid atherosclerotic plaque (CAP). The blood samples were collected from the People's Hospital of Guangxi Zhuang Autonomous Region from March 2016 to May 2018. All patients signed informed consent forms, and the study was approved by the Ethics Committee of the People's Hospital of Guangxi Zhuang Autonomous Region.

### 2.2. Blood Sample Collection

Blood samples were collected from each subject at 6-7 A.M. on the day following admission. All the samples were collected in sodium heparin vacutainers and quickly taken to the laboratory. The samples were then centrifuged as at 4000 × *g* for 20 min to obtain the plasma, which was subsequently frozen and preserved in liquid nitrogen until use. The blood samples were collected by nurses with more than 8 years of experience, and the time from blood samples collection to plasma storage was no longer than 45 min.

### 2.3. Detection of Plasma Sesn1, Sesn2, and Sesn3 Levels

The Sesn1, Sesn2, and Sesn3 enzyme-linked immunosorbent assay (ELISA) kits were purchased from Cloud-Clone Corp. (USA). The performance of these ELISA kits was tested. The lower detection limit of Sesn1 was approximately 10.5 ng/mL, the intra-assay and interassay coefficients of variation were <5% and <7%, respectively, the recovery range was between 90.5 and 115.2%, and the sensitivity was approximately 1.0 ng/mL. The lower detection limit of Sesn2 was approximately 0.5 ng/mL, the intra-assay and interassay coefficients of variation were <4% and <8%, respectively, the recovery range was between 93.4 and 110.5%, and the sensitivity was approximately 0.2 ng/mL. For Sesn3, the lower detection limit was approximately 3.2 ng/mL, the intra-assay and interassay coefficients of variation were <4.5% and <9.5%, respectively, the recovery range was between 92.5 and 116.6%, and the sensitivity was approximately 0.5 ng/mL.

Plasma samples were taken from liquid nitrogen and defrosted at 4°C. The plasma levels of Sesn1, Sesn2, and Sesn3 were detected using the respective ELISA kits, according to the manufacturer's instructions. All tests were performed in duplicate.

### 2.4. Statistical Analysis

Data regarding the enumeration variables were expressed as the mean ± standard deviation (SD). Student's *t*-test was performed to compare the difference between the two groups, and one-way ANOVA, followed by the LSD test, was performed for multiple comparisons between more than two groups. The categorical variables are presented as counts (percentages) and compared with the chi-square test. The correlations between Sesn2 levels and blood pressure (including SBP and DBP) were calculated using Spearman's correlation analysis. The simple linear regression analysis and subsequent multiple linear regression analysis were performed to investigate the role of Sesns in the presence of CAP. All the data were analyzed using the SPSS 23.0 software, and *p* < 0.05 was considered statistically significant.

## 3. Results

### 3.1. The Differences in the Clinical Characteristics of Each Group

Compared with the control group, hypertension patients exhibited higher SBP, DBP, high-density lipoprotein cholesterol (HDL-C), creatinine (CREA), C-reactive protein (CRP), homocysteine (Hcy), and angiotensin II (Ang II). No other differences in clinical characteristics were identified between the two groups, including age, male gender, smoking, the duration of hypertension (history), heart rate (HR), body mass index (BMI), total cholesterol (TC), total triglycerides (TG), low-density lipoprotein cholesterol (LDL-C), fasting glucose (Glu), and hemoglobin a1c (HbA1c). In addition, the SBP, DBP, CREA, CRP, Hcy, Ang II, and CAP percentages were shown to gradually increase between the grade I, grade II, and grade III groups. The history in the grade II and grade III groups was higher than that in the grade I group, while the level of high-density lipoprotein cholesterol (HDL-C) in the grade III group was lower than that of the other two groups. The clinical characteristics of each group are listed in Table 1.

In addition, the dipper group and nondipper group exhibited higher SBP, DBP, CREA, CRP, Hcy, and Ang II when compared to the control group. Moreover, SBP and CRP were higher in the dipper group when compared with the nondipper group, while DBP, HR, CREA, and CAP percentages exhibited the opposite trend. The clinical characteristics of the dipper and nondipper groups are listed in Table 2.

### 3.2. Plasma Sesn1, Sesn2, and Sesn3 Levels Were Increased in Hypertension Patients

**Figure 1:**
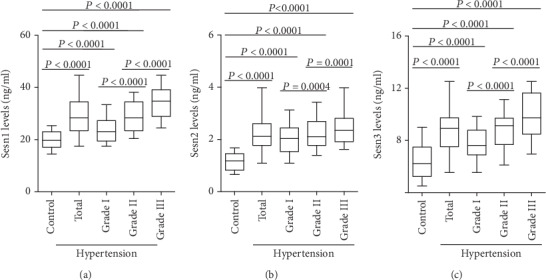
Circulating Sesn levels in normotensive subjects (control) and hypertension patients. (a) Plasma Sesn1 levels in the control group (*n* = 100), total hypertension group (*n* = 400), grade I group (*n* = 140), grade II group (*n* = 180), and grade III group (*n* = 80) were measured using ELISA kits. (b) Plasma Sesn2 levels were detected in each group. (c) Plasma levels of Sesn3 were determined in the four groups.

Plasma Sesn1 levels were significantly increased in hypertension patients when compared with the control group and gradually increased between the grade I, grade II, and grade III groups (Figure 1(a)). Plasma Sesn2 and Sesn3 exhibited similar trends to Sesn1 (Figures 1(b) and 1(c)). Furthermore, plasma Sesn1, Sesn2, and Sesn3 levels were positively correlated to both SBP and DBP in hypertension patients. The *r* values and *p* values are listed in Table 3.

### 3.3. Nondipper Hypertension Patients Exhibited Higher Sesn1, Sesn2, and Sesn3 Levels

**Figure 2:**
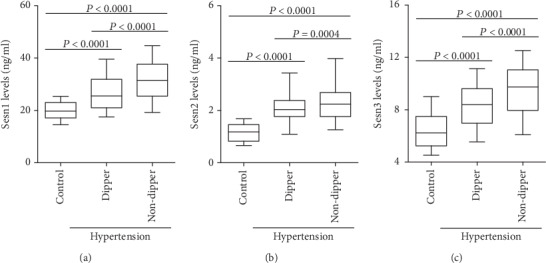
Plasma Sesn levels in dipper hypertension and nondipper hypertension patients. The plasma (a) Sesn1, (b) Sesn2, and (c) Sesn3 levels in the control group (*n* = 100), dipper hypertension group (*n* = 240) and nondipper hypertension group (*n* = 160) were analyzed by ELISA.

According to the results of the ambulatory blood pressure monitoring, the hypertension patients were divided into the dipper and nondipper groups. The ELISA results revealed that plasma Sesn1, Sesn2, and Sesn3 levels gradually increased between the dipper and nondipper groups (Figures 2(a)–2(c)).

### 3.4. Effects of Clinical Characteristics on Plasma Sesn1, Sesn2, and Sesn3 Levels

Subjects were divided into two groups based on the median of several characteristics, such as HDL-C, CREA, CRP, Hcy, Ang II, smoking status, CAP, and medication use. The results showed that smokers and patients with CAP and higher Ang II (≥Median) exhibited higher plasma Sesn1, Sesn2, and Sesn3 levels. HDL-C, CREA, CRP, Hcy, and use of ACEI/ARB, *β*-blockers, CCB, and diuretics did not affect Sesn expression. Sesn1, Sesn2, and Sesn3 levels in each group are listed in Table 4.

### 3.5. Sesn1, Sesn2, and Sesn3 Were Negatively Correlated with CAP Occurrence

To determine the effects of Sesns on CAP occurrence, Sesn1, Sesn2, and Sesn3 levels, as well as smoking status, SBP, DBP, LDL-C, Glu, CRP, Hcy, and Ang II were used to perform simple linear regression analysis. The results revealed that Sesn1, Sesn2, Sesn3, smoking, SBP, DBP, CRP, Hcy, and Ang II showed a trend. These variables were further used to perform a multivariate linear regression analysis. The results showed that Sesn1, Sesn2, and Sesn3, respectively, were independently associated with the presence of CAP. The *β* value, 95% CI of *β*, and *p* values are listed in Table 5.

## 4. Discussion

In the present study, the plasma levels of the antioxidant proteins Sesn1, Sesn2, and Sesn3 in hypertension patients were detected. The results of our study revealed that Sesn1, Sesn2, and Sesn3 levels were significantly increased in hypertension patients when compared with the normotensive subjects. A further analysis showed a positive correlation between plasma Sesn levels and blood pressure values, including SBP and DBP. In addition, we identified a variety of variables, including smoking status, Ang II, and CAP, which could promote Sesn expression. Furthermore, the results of the multiple linear regression analysis suggested that Sesn1, Sesn2, and Sesn3 levels were negatively correlated with the presence of CAP. Our study is the first to show the increased expression of Sesns in hypertension patients, which may have a protective effect against the development of CAP.

Elevated Sesn levels have been widely reported in a variety of diseases. In a previous study, circulating Sesn1, Sesn2, and Sesn3 were reported to be increased in patients with coronary artery diseases, while the amplitude of the increase in Sesn levels was closely related to the degree of stenosis and oxidative stress in CAD patients [15]. In another study of human aortic dissection, plasma Sesn2 levels were found to be positively correlated with MDA levels and negatively correlated with SOD levels [16]. Considering that oxidative stress is the most important factor that affects Sesn expression, we suspected that the level of increase in Sesn expression may reflect the severity of the oxidative stress. Indeed, treatment with Ang II and oxidized low-density lipoprotein, which were shown to induce intense oxidative stress, increased Sesn2 mRNA levels in macrophages in a dose-dependent manner [17, 23, 24]. These findings further support our hypothesis that there is a strong association between oxidative stress levels and Sesn expression and that they can influence each other.

In this study, we found that plasma Sesn levels were increased in patients with hypertension and were positively correlated with the value of blood pressure. These results suggest that there are different degrees of oxidative stress imbalance among hypertensive patients. Moreover, intense oxidative stress may be one of the key players in the pathogenesis of hypertension. As such, it may be useful to pay attention to the dynamic changes in both blood pressure and oxidative stress.

In healthy subjects, blood pressure variability over a 24 h period presents as a double peak and a valley; namely, the highest blood pressure appears in the morning and afternoon, while the lowest blood pressure appears at night. This is due to a drop in sympathetic nerve excitability in the evenings and is called the dipper blood pressure. In some patients with hypertension, this blood pressure trend is disrupted and the blood pressure at night is higher than that in the morning, which is called nondipper hypertension. Since nondipper hypertension is not easy to detect and the damage to target organs is significantly higher than that of dipper hypertension, nondipper hypertension is more harmful to patients [25, 26]. There is evidence that sympathetic excitation promotes the release of large amounts of peroxidation-related substances [27]. In our study, we found a significant increase in Sesn levels in nondipper hypertension patients, which represents excessive oxidative stress. One possible reason is that sympathetic hyperactivity may mediate the formation of nondipper hypertension by enhancing oxidative stress and that reducing the oxidative stress levels may be beneficial to the treatment of nondipper hypertension and the reduction of target organ injury mediated by this condition.

Previously, we revealed that Sesns are involved in the regulation of hypertension, mainly via its antioxidant effects. In an early study, Yang et al. reported that silencing Sesn2 aggravated oxidative stress and elevated the blood pressure in dopamine D2 receptor (D2R) knockout-induced hypertension mice [28]. In a recent study, Xiao et al. found that Sesn2 promoted the expression of the nuclear factor erythroid-2-related factor-2 (Nrf2), which then alleviated Ang II-induced smooth muscle cells apoptosis [17]. While we found that Sesn levels increase in the oxidative stress environment of hypertension, the conclusion of the studies mentioned above seems to contradict our results. One possible explanation is that Sesn levels increase in response to oxidative stress and that Sesns act as antioxidants that protects organs and tissues from damage.

Smoking, angiotensin, atherosclerosis, and a variety of antihypertensive drugs are closely related to oxidative stress [29–31]. To determine whether these factors affect Sesn expression, the hypertension patients were divided into 2 groups. The results showed that patients with higher Ang II, smoking, and CAP exhibited higher plasma Sesn1, Sesn2, and Sesn3 levels, while other factors and the use of antihypertensive drugs did not affect Sesn expression. To investigate the role of Sesns in the occurrence of CAP, multivariate linear regression analysis was performed. The results revealed that Sesn1, Sesn2, and Sesn3 were independently associated with the presence of CAP. These results suggest that Sesns may be useful in preventing and delaying the development and progression of atherosclerosis. However, this hypothesis requires further confirmation in animal models.

In conclusion, we found that plasma Sesn1, Sesn2, and Sesn3 levels were increased in hypertension patients and positively correlated to the blood pressure values. Nondipper hypertension patients exhibited higher plasma Sesn1, Sesn2, and Sesn3 levels when compared to dipper hypertension patients. Moreover, Sesns may be involved in hypertension or CAP through the regulation of oxidative stress. As such, Sesns may be a novel target for the treatment and prevention of hypertension and CAP. However, there were some limitations in the present study. The oxidative stress markers such as SOD and MDA levels were not quantified in the patients included in the study, and the correlation between SOD, MDA, and Sesns was not analyzed. Moreover, while there is a strong relationship between inflammatory response and oxidative stress, Sesn expression and oxidative stress levels can affect each other. However, we did not explore the potential link between them.

## Figures and Tables

**Table 1:** Clinical characteristics in each group.

Characteristics	Control	Hypertension			
		Total	Grade I	Grade II	Grade III
Age (years)	48.2 ± 9.4	47.8 ± 9.9	48.8 ± 10.3	47.1 ± 9.4	47.6 ± 9.6
Male (*n*, %)	66 (66.0)	287 (71.8)	102 (72.9)	126 (70.0)	59 (73.4)
Smoking (*n*, %)	33 (33.0)	145 (36.3)	47 (33.6)	62 (34.4)	36 (45.0)
History (years)	—	10.1 ± 7.4	11.4 ± 8.6	9.4 ± 7.7^$^	9.5 ± 7.1^$^
SBP (mmHg)	111 ± 11	165 ± 18^∗^	147 ± 12^∗,#^	169 ± 15^∗,$^	189 ± 20^∗,#,$,&^
DBP (mmHg)	74 ± 8	95 ± 10^∗^	86 ± 9^∗,#^	98 ± 10^∗,$^	104 ± 11^∗,#,$,&^
HR (HR)	68 ± 6	70 ± 9	71 ± 9	68 ± 8	72 ± 10
BMI (kg/m^2^)	24.5 ± 3.1	24.5 ± 3.2	24.3 ± 2.9	24.7 ± 3.1	25.3 ± 3.5
TC (mmol/L)	4.5 ± 0.8	4.4 ± 0.9	4.6 ± 1.0	4.3 ± 0.8	4.5 ± 0.9
TG (mmol/L)	1.8 ± 1.1	1.8 ± 1.2	2.0 ± 1.3	1.7 ± 1.2	1.9 ± 0.9
HDL-C (mmol/L)	1.3 ± 0.3	1.1 ± 0.3^∗^	1.1 ± 0.3^∗^	1.2 ± 0.3	1.0 ± 0.3^∗,&^
LDL-C (mmol/L)	2.6 ± 0.7	2.7 ± 0.8	2.8 ± 0.8	2.5 ± 0.7	2.9 ± 0.9
Glu (mmol/L)	5.4 ± 0.6	5.5 ± 0.8	5.7 ± 0.8	5.3 ± 0.9	5.5 ± 0.8
HbAlc (%)	4.7 ± 0.8	4.5 ± 0.8	4.4 ± 0.6	4.6 ± 0.8	4.5 ± 0.9
CRRA (*μ*mol/L)	71 ± 11	93 ± 20^∗^	81 ± 15^∗,#^	94 ± 19^∗,$^	111 ± 24^∗,#,$,&^
CRP (mg/L)	0.9 ± 0.6	6.6 ± 3.6^∗^	3.9 ± 1.6^∗,#^	7.5 ± 3.8^∗,#,$^	9.3 ± 4.4^∗,#,$,&^
Hcy (pg/mL)	10.6 ± 3.4	15.3 ± 5.8^∗^	11.7 ± 4.6^∗,#^	15.9 ± 5.2^∗,$^	20.2 ± 7.3^∗,#,$,&^
Ang II (pg/mL)	34.9 ± 14.1	57.5 ± 24.1^∗^	45.5 ± 19.5^∗,#^	59.9 ± 24.4^∗,$^	72.9 ± 29.1^∗,#,$,&^
CAP	—	200 (50.0)	52 (37.1)^#^	93 (51.7)^$^	55 (68.8)^#,$,&^
Medical treatments (*n*, %)					
ACEI/ARB	—	244 (61.0)	70 (50.0)^#^	111 (61.1)	63 (78.8)^#^
*β*-Blocker	—	179 (44.8)	52 (37.1)	83 (46.1)	44 (55.0)
CCB	—	338 (84.5)	109 (77.9)^#^	159 (88.3)	70 (87.5)
Diuretics	—	244 (41.3)	30 (21.4)^#^	74 (41.1)^$^	61 (76.3)^#,$,&^

History: the duration of hypertension; SBP: systolic blood pressure; DBP: diastolic blood pressure; HR: heart rate; BMI: body mass index; TC: total cholesterol; TG: total triglycerides; HDL-C: high-density lipoprotein cholesterol; LDL-C: low-density lipoprotein cholesterol; Glu: fasting glucose; HbA1c: hemoglobin a1c; CRP: C-reactive protein; CREA: creatinine; Hcy: homocysteine; Ang II: angiotensin II; CAP: carotid atherosclerotic plaque; ACEI: angiotensin-converting enzyme inhibitor; ARB: angiotensin receptor blocker; CCB: calcium channel blocker. ^∗^*p* < 0 05 vs. the control group, ^#^*p* < 0 05 vs. the total group, ^$^*p* < 0 05 vs. the grade I group, and ^&^*p* < 0 05 vs. the grade II group.

**Table 2:** Clinical characteristics in the dipper group and nondipper group.

Characteristics	Control	Hypertension	
		Dipper	Nondipper
Age (years)	48.2 ± 9.4	48.0 ± 10.1	47.5 ± 8.9
Male (*n*, %)	66 (66.0)	167 (69.6)	120 (75.0)
Smoking (*n*, %)	33 (33.0)	78 (32.5)	77 (48.1)^∗,#^
History (years)	—	9.9 ± 7.5	10.5 ± 8.8
SBP (mmHg)	111 ± 11	167 ± 15^∗^	163 ± 11^∗,#^
DBP (mmHg)	74 ± 8	94 ± 9^∗^	96 ± 10^∗^
HR (HR)	68 ± 6	71 ± 9^∗^	68 ± 8^#^
BMI (kg/m^2^)	24.5 ± 3.1	24.5 ± 2.8	25.0 ± 3.4
TC (mmol/L)	4.5 ± 0.8	4.4 ± 0.8	4.5 ± 0.9
TG (mmol/L)	1.8 ± 1.1	1.8 ± 1.1	1.9 ± 1.2
HDL-C (mmol/L)	1.3 ± 0.3	1.1 ± 0.3^∗^	1.2 ± 0.4
LDL-C (mmol/L)	2.6 ± 0.7	2.6 ± 0.7	2.8 ± 0.9
Glu (mmol/L)	5.4 ± 0.6	5.4 ± 0.7	5.6 ± 0.9
HbAlc (%)	4.7 ± 0.8	4.5 ± 0.7	4.5 ± 0.9
CRRA (*μ*mol/L)	71 ± 11	90 ± 18^∗^	97 ± 23^∗,#^
CRP (mg/L)	0.9 ± 0.6	7.4 ± 4.6^∗^	5.4 ± 2.9^∗,#^
Hcy (pg/mL)	10.6 ± 3.4	15.1 ± 5.7^∗^	15.6 ± 6.4^∗^
Ang II (pg/mL)	34.9 ± 14.1	57.1 ± 24.7^∗^	58.1 ± 26.2^∗^
CAP	—	109 (45.4)	91 (56.9)^∗^
Medical treatments (*n*, %)			
ACEI/ARB	—	146 (60.8)	98 (61.2)
*β*-Blocker	—	101 (40.1)	78 (48.8)
CCB	—	200 (83.3)	138 (86.3)
Diuretics	—	141 (58.8)	101 (63.1)

^∗^ *p* < 0 05 vs. the control group; ^**#**^*p* < 0 05 vs. the dipper group.

**Table 3:** Correlation between blood pressure and the Sesn levels.

Characteristic	SBP		DBP	
	*r* value	*p* value	*r* value	*p* value
Sesn1	0.3262	<0.0001	0.4198	<0.0001
Sesn2	0.4511	<0.0001	0.3309	<0.0001
Sesn3	0.2987	<0.0001	0.4419	<0.0001

**Table 4:** Sesn levels according to characteristics in hypertensive patients.

Characteristics	Factors	Sesn1 (ng/mL)	Sesn2 (ng/mL)	Sesn3 (ng/mL)
Smoking	Yes	30.6 ± 7.9	2.49 ± 0.81^∗^	9.44 ± 2.8^∗^
	No	27.4 ± 5.1	2.0 ± 0.51	8.1 ± 1.6

HDL-C	≥Median	29.1 ± 6.8	2.19 ± 0.68	8.8 ± 1.7
	<Median	28.9 ± 7.4	2.30 ± 0.64	8.7 ± 1.6

CREA	≥Median	28.8 ± 5.7	2.24 ± 0.69	8.8 ± 1.9
	<Median	29.2 ± 7.6	2.25 ± 0.59	8.8 ± 1.6

CRP	≥Median	30.1 ± 6.7	2.39 ± 0.77	9.0 ± 2.0
	<Median	26.9 ± 6.1	2.10 ± 0.49	8.6 ± 1.5

Hcy	≥Median	28.8 ± 5.9	2.20 ± 0.62	8.7 ± 1.4
	<Median	29.2 ± 6.5	2.29 ± 0.68	8.8 ± 1.9

Ang II	≥Median	36.6 ± 7.2^∗^	2.88 ± 0.79^∗^	10.7 ± 2.4^∗^
	<Median	21.4 ± 5.9	1.95 ± 0.54	6.8 ± 1.4

CAP	Yes	32.8 ± 6.9^∗^	2.69 ± 0.75^∗^	9.8 ± 2.1^∗^
	No	25.1 ± 6.8	1.80 ± 0.66	7.7 ± 1.3

ACEI/ARB	Yes	29.1 ± 5.9	2.21 ± 0.49	8.7 ± 1.2
	No	28.8 ± 7.1	2.28 ± 0.76	8.8 ± 1.5

*β*-Blocker	Yes	28.6 ± 7.3	2.19 ± 0.66	8.6 ± 1.1
	No	29.3 ± 7.7	2.30 ± 0.74	8.9 ± 1.8

CCB	Yes	29.1 ± 7.9	2.24 ± 0.61	8.8 ± 1.6
	No	28.8 ± 7.1	2.24 ± 0.77	8.8 ± 2.0

Diuretics	Yes	28.9 ± 5.5	2.27 ± 0.55	8.7 ± 1.5
	No	29.1 ± 7.9	2.22 ± 0.81	8.8 ± 2.4

^∗^ *p* < 0 05.

**Table 5:** Association between plasma Sesns and the presence of CAP was assessed by simple linear regression analysis and subsequent multiple linear regression analysis.

Variables	Simple linear			Multiple linear		
	*β*	95% CI	*p* value	*β*	95% CI	*p* value
Sesn1	-0.415	-0.636 to -0.194	<0.001	-0.218	-0.304 to -0.132	0.011
Sesn2	-0.556	-0.794 to -0.318	<0.001	-0.301	-0.408 to -0.194	<0.001
Sesn3	-0.487	-0.715 to -0.259	<0.001	-0.247	-0.342 to -0.152	0.009
Smoking	0.339	0.154 to 0.524	0.002	0.144	0.086 to 0.201	0.039
SBP	0.357	0.167 to 0.547	0.009	0.157	0.094 to 0.220	0.068
DBP	0.289	0.115 to 0.463	0.011	0.112	0.052 to 0.172	0.104
LDL-C	0.109	0.062 to 0.156	0.198			
Glu	0.212	0.104 to 0.320	0.239			
CRP	0.117	0.071 to 0.163	0.019	0.046	-0.017 to 0.109	0.042
Hcy	0.098	0.047 to 0.149	0.026	0.087	0.022 to 0.152	0.672
Ang II	0.614	0.383 to 0.845	<0.001	0.356	0.455 to 0.257	<0.001

## Data Availability

We declare that the materials described in the manuscript, including all relevant raw data, will be freely available to any scientist wishing to use them for noncommercial purposes, without breaching participant confidentiality.
